# Data sharing for citizen-driven repair and reuse of electronics and electrical equipment

**DOI:** 10.1016/j.dib.2026.112895

**Published:** 2026-05-28

**Authors:** Umair ul Hassan, Prateek Paul, Muhammad Sohaib Ayub, Kashif Shaheed, Long Hoang

**Affiliations:** a Insight Research Ireland Centre for Data Analytics, University of Galway, Ireland; b J.E. Cairnes School of Business and Economics, University of Galway, Ireland; c Data Science Institute, University of Galway, Ireland

**Keywords:** Data sharing, Data space, Repairability, Circular economy, E-waste

## Abstract

A key objective of the European data strategy is to establish a common European Green Deal data space, which aims to address all European Green Deal related topics, including biodiversity, climate action, clean energy, sustainable mobility, and circular economy, by facilitating secure and interoperable data sharing across these domains. Emphasizing the Right to Repair directive, the development of a secure data sharing architecture, along with the necessary legal instruments and governance structures, is critical for success. However, one of the primary barriers to achieving this goal is the engagement of stakeholders in the processes of data collection, curation, sharing, and utilization. In this paper, we present our implementation strategy of a data sharing platform specifically designed for the repair and reuse of electrical and electronic equipment. This platform was developed as part of the Sharepair project, which seeks to reduce Waste Electrical and Electronic Equipment (WEEE) by scaling citizen-driven repair initiatives through the use of digital tools. Additionally, we offer recommendations for enhancing the European online repair platform based on our findings and experiences.

## 1 Background

Responsible consumption and production are one of the UN’s Sustainable Development Goals [1]. Despite growing awareness about the need to shift towards more sustainable patterns of consumption, waste from consumer goods in Europe and in other developed economies remains high. Therefore, there is an urgent need to prevent this planned o accumulation of waste and decrease over-consumption of equipment [2]. It is particularly so for *waste electrical and electronic equipment* (WEEE) which is one of the fastest-growing waste streams in Europe, and in 2022, out the 14.4 million tonnes of *electrical and electronic equipment* (EEE) put on market only 5 million tonnes was collected [3]. Consequently, the “Right to Repair” movement has gained significant momentum in Europe over past few years, and it has resulted some crucial legislation such as the directive on common rules promoting the repair of goods [4]. This legislation is aimed at increasing the sustainable consumption by increase in repair and reuse of goods.

From a resource-efficiency perspective, reuse of EEE products and components is better than recycling, because recycling often still requires a significant amount of new materials and energy to manufacture new products. Reuse, repair and refurbishment thus have the potential to be powerful levers in the transition to a circular economy, in which products are designed for longer use through sharing and repair before recycling. In general, there are three setting which the repairs of EEE products takes place [5]: by commercial repairers; on a voluntary basis, e.g., in repair cafés and at events; or by consumers performing repairs themselves, i.e., DIY repairs. Europe is home to many citizen-driven initiatives that focus on repair and reuse of products. For instance, the founding members of the *Open Repair Alliance* (ORA) are all based in Europe and represent a community of 1500+ repair clubs and reuse facilities [6]. Yet, as highlighted by a European Commission study on the socioeconomic impacts of enhanced product reparability, repairers often express frustration over limited access to original spare parts, technical information, diagnostic software, and training [7]. Moreover, the impact of such citizen-driven repair and reuse initiatives is still limited; furthermore, these initiatives are dispersed and lack critical mass. Although each repair event helps to avoid e-waste, approximately 40% of the products brought to a repair event are not repaired, often due to missing information or a lack of spare parts or components.

Search frictions faced by people involved in repair, reuse, or refurbishment of EEE present a practical challenge towards e-waste prevention [8,9]. The lack of technical information and knowledge significantly hinders electronic product repairers, as many manufacturers restrict access to essential repair manuals, diagnostic tools, and proprietary software, which are often only available to authorized sources [9]. This limitation leaves repairs without the necessary resources to accurately diagnose and fix devices, leading to longer repair times and potential errors. Additionally, the rapid evolution of technology creates skill gaps, as many repairers lack formal training on newer models and complex issues [9]. Without reliable online resources and community support, repairers struggle to stay updated, ultimately affecting consumer trust in their services and contributing to a culture of disposability.

## 2 Digital Tools to Support Citizen Repairers

Funded by the European Commission under the Interreg North-West programme, the Sharepair project aimed to explore how digital technologies and tools can be employed to support citizen repair initiatives for reducing waste electrical and electronic equipment (WEEE) [10]. To achieve this, the project developed an integrated approach to scaling up the citizen repair initiatives across several cities in North-West Europe. The approach focused on developing a digital infrastructure consisting of following digital technologies: (i) a support tool for repairers in repair clubs and repair centres (ii) a guidance tool for consumers in households, (iii) a tool for mapping repair facilities and repair services in the participating regions and cities, with a particular focus on local commercial firms with high-quality services, (iv) a tool for collecting and sharing open-source designs for 3D printing of spare parts, and most importantly (v) an integrated data platform that gathers all the data captured by the above. The Sharepair ran between 2019 and 2023 with partner organization ranging from city administrations, repair collectives, and research institutions in Belgium, Netherlands, United Kingdom, Ireland, and Germany. Following this project, more projects were funded by the European Commission to support the repair activities for electronics projects [11]. For instance, the RERper project focuses on aiding SMEs in the repair sector, enhancing repair skills, and promoting sustainable consumer choices, while rethinking traditional business models, reemphasizing human value, and reassessing behaviours to transition towards a circular economy [12]. On the other side, the Circular WEEP project aims to design and evaluate policies related to reduction, repair, and reuse of electronic products [13]. These projects highlight the continuing interest and support of research and innovation activates in the product repairability. More importantly, through a recent directive, the EU is establishing a *European Online Repair Platform* (EORP) that will connect stakeholder and provide harmonized information about products, their repairability, local repair services and community initiative [14]. Such a platform will essentially serve as the data space for sharing repairability related data across Europe.

As a precursor to the EORP, the Sharepair project organized workshops with technical and non-technical stakeholders to discuss existing data sources on device repairs collected by repair communities. These discussions revealed that efforts to collect and make repair data openly available are still in their early stages. The main sources of openly available datasets and data sources are the repositories maintained by ORA and iFixit^1^. The ORA shares data on products and their repairs from member organizations, including Anstiftung Foundation, Fixit Clinic, iFixit, Repair Café, and The Restart Project^2^; however, the metadata is only available in HTML format. On the other hand, iFixit data is the largest in terms of device and repair guide numbers and is available via JSON API with HTML metadata. Additionally, community repair projects such as The Restart Project, Repair Café, and Reparatur Initiativen^3^ provide openly available data in HTML format. The Sustainable Design Center eV also openly shares 3D designs of product parts, enabling users to print parts when they are not readily available. Table 1 summarizes the existing openly available datasets and data sources.

**Table 1:** List of existing data sources for open repair data.

Publisher	Description	Entities in data	Format
*Open Repair Alliance*	Data collected by multiple repair organizations according to Open Repair Data Standard	Products, Repairs, Sessions, Providers, Repair Records	CSV, HTML
*iFixit*	Wiki of device repairs	Devices, Repair Guides, Stories	JSON, HTML
*Restart Project*	Developers of Fixometer and Restarters.net	Devices, Events, Repairs, Groups	CSV, HTML
*Repair Café International Foundation*	Online platform for repair cafés across the globe	Products, Repairs, Repair Cafés, Events	HTML
*Reparatur Initiativen*	Online platform for repair initiatives in Germany	Repair Cafés, Events	HTML
*Sustainable Design Center eV*	Platform for 3D printable parts	3D Models, Text, Pictures, Lists, PDFs, Instructions, Presentation Lists, Slides	STL, STEP, PDF, TXT, HTML, JPG

### 2.1 Open repair data standard

**Fig. 1:**
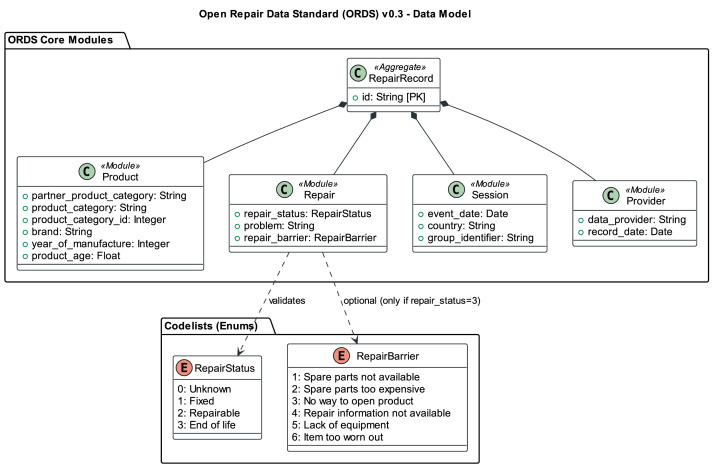
Data model of the open repair data standard v0.3 [15].

Harmonization of repair information across member states, companies, and products is a key objective of the European repair directive [14]. This should in theory translate in interoperability of repair data at the time of collection, exchange, and dissemination. Towards this goal, the ORA has taken lead in standardizing the semantics of repair data collected by repair community by creating the *Open Repair Data Standard* (ORDS) [15]. As detailed in Fig. 1, the current schema of the ORDS covers four key entities: *product, repair, session*, and *provider* [15]. The ORDS is designed as a bridging standard to interlink and harmonise data from repairs activities conducted by community repairers and individuals. Data based on ORDS can be further supplemented with existing open ontologies and vocabularies from W3C and Schema.org to describe products and their associated repairability information. For instance, its fields map naturally to the Schema.org vocabulary (specifically with entities like *RepairAction, Product*, and *Brand*). There is ongoing discussion in the circular economy community about creating a JSON-LD (Linked Data) serialization for ORDS to make it fully compatible with the Semantic Web.

Further linkages between different datasets can be achieved by describing the shared data using well-known and emerging standards. The IEEE oManual is an open XML-based standard for creating and distributing structured manuals with rich multimedia such as repair guides and how to instructions [16]. The ORDS complements this with details of repair sessions and their results. The IOP-EC (Open Electronics Design Data) is an initiative by the Internet of Production Alliance and KitSpace that aims to create a new standard for open-sources documentation for electronics design [17]. The ORDS provides feedback loop for data for these designs in terms of most common failed products and components. In terms of e-waste reporting, the ORDS product categories are mapped to the relevant EU’s WEEE categories. This allows data from repair activities to be aggregated and analysed. Furthermore, the ORDS can be considered a bottom-up sources of data for the upcoming *Digital Product Passport* (DPP) [18]. While the DPP will likely contain top-down data from manufacturers (manuals, material lists), ORDS provides the post-consumer data (durability, common points of failure) that completes the product’s lifecycle story. Within the Sharepair project, ORDS data has been used to connect repairable products to 3D-printing repositories, allowing a record in ORDS to trigger the discovery of a printable replacement part. Given this central role of the ORDS, it should be further promoted and extended further as a standard within the European open data models. This aligns with the European Data Strategy, particularly the Green Deal Data Space [[19], [20], [21]], which aims to create interconnected cloud infrastructures and common European data spaces. The strategy emphasizes interoperability, with data providers ensuring minimal interoperability mechanisms.

### 2.2 Open repair data platform

By adopting and expanding the ORDS, the Sharepair project focused on prototyping of an *Open Repair Data Platform* (ORDP) to support digital tools for citizen-led repair events and initiatives [22,23]. Within the Sharepair project, a suite of tools worked together to provide comprehensive repair support to repairers. The tools combined user data to enhance repair activities through feedback on: volume and categories of products repaired, success rates and barriers, and the impact on material use and climate. The main tools in Sharepair included:

• **Logging/Registration Tools**: Based on the ORDS, these tools collected logging data from repair cafés and extended it in breadth and depth. Best practices collected in wikis, tips, and tricks were fed back into an interactive repair application to support self-repair.
• **3DP Design Platform**: This platform collected and shared the best available 3D printing designs for repair, along with advice on material usage, training, and more.
• **Citizen Repair Guide**: Developed in collaboration with a citizen science project, this guide created a decision tree that connects to other digital modules, providing a comprehensive solution approach for citizens.
• **Repair Service Mapping**: This tool mapped repair services, including professional services and spare part platforms with buy, recycle, and print options.
• **Manuals and Tool Libraries**: iFixit manuals and other resources, as well as tool libraries, were connected to the decision trees in the repair guidance, providing users with a wealth of information and support.

These tools worked together to create a robust repair support infrastructure that empowered users to repair and maintain products effectively.

## 3 Sharepair Data Strategy

The Sharepair project enhances the growing data power of citizen-oriented repair by integrating data from community repair initiatives, city repair communities, and consumer organizations. To achieve a strategy for scaling up citizen repair with digital tools, project partners and associated organization pooled their data into an open repair data platform . The ORDP was a European-level initiative that aggregated relevant repair data from a citizen's perspective, and it was first project aimed towards creation of a European data space for repairs of electronic and electrical products [22]. At its core, the ORDP was designed to be a distributed system based on several nodes deployed using cloud services thus providing a data space connected to dedicated digital repair tools, serving as an open data resource for open-source tools that support citizen repair.

### 3.1 ORDP technical architecture

**Fig. 2:**
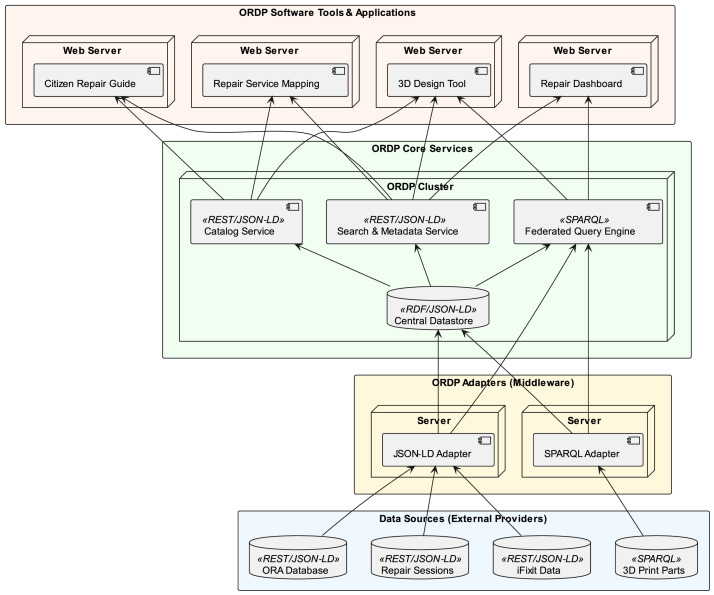
A layered architecture for open repair data platform (ORDP) and digital tools of the Sharepair project.

The data architecture of the ORDP encompasses all tools and data sources that can provide integrated solutions for citizen repair, as shown in Fig. 2. The platform's strategy is built on openness and sharing to maximize impact. The Sharepair project's data structure, based on the ORDS, serves as a pilot for the EORP. The ORDP was envisioned as a public resource, managed for the common good, with data management guided by European values of personal integrity and societal benefit. Citizens retain ownership of the data they produce. A network of Smart&Circular cities were engaged to play a key role in managing the platform, ensuring that the data is used to benefit society as a whole.

A prototype ORDP was developed as part of the Sharepair project, and it provides a blueprint for future similar efforts. From the architectural point of view, the ORDP consisted of four layers of nodes and components while following a linked data approach [22]:

1. **Data Sources**: These include repositories of product data, repair data, parts data, and more that were external to the ORDP. They form the core data sources of the shared data space for product repairs. A data provider can add or remove a data sources from the shared data space, where addition of data source entailed some ``cost'' such as registration with the ORDP via its catalog; conformance to ORDS and related standard data models; and publishing of data in specific formats like JSON-LD based on Linked Open Data principles [24,25]. At minimum, each data source was required to provide a REST/JSON-LD endpoint, with SPARQL endpoint as optional. All such interfaces were required to include metadata and data quality information using W3C vocabularies such as DCAT, VoID, and DQV.
2. **ORDP Adapters**: These were technical interfaces that served as middleware services to provide access to the underlying data. Adapters validated the JSON-LD payload of data sources; provided mapping to ORDS and related data models; exposed a unified REST/JSON-LD and SPARQL APIs for data consumers; and enabled the measurement of a data source's integration level, as showed in Table 2, based on a 5-star scheme of Open Linked Data [24,25]. For example, a dataset with a SPARQL interface is more integrated than one exposed only through a REST API with a fixed set of methods.

**Table 2:** Data source integration‑level criteria and associated functionalities.

★	No SPARQL endpoint, only REST upload.
★★	REST upload and static JSON‑LD schema.
★★★	SPARQL endpoint with basic query support.
★★★★	SPARQL and real‑time data push via WebSocket.
★★★★★	Full semantic integration, including reasoning support.
3. **ORDP Core Services**: These services formed the backbone of the ORDP, allowing data sources to be visible, understandable, queryable, interoperable, integrable, searchable, and monitorable. These services were implemented following a hybrid approach. Data from providers was ingested in a centralized datastore using the JSON-LD and SPARQL based adapters. In turn, this data was exposed using a data catalog that enabled data providers to register and remove data sources, and it enabled data discovery by providing information to data consumers on how to access and map the data. In conjunction with catalog service, the search and metadata services provided semantic search over description of data sources and exposed metadata about each data source in machine readable format. Finally, a federated query engine that allowed semantic queries across all data sources on demand.
4. **ORDP Software Tools and Applications**: These applications consume the data from ORDP core services and adapters to provide user facing tools and services. For instance, the developer of a repair dashboard can use the catalog service to understand a data source's format and schema, and then develop a web-based dashboard that queries the data directly from adapters or through federated query engine to calculate statistics over products, repairs, and faults. Similarly, a commercial repairer can use the 3D Design Tool to find the printable 3D design of an old part of faulty washing machine.

### 3.2 ORDP governance and business models

Sharepair’s digital infrastructure was intricately linked with the physical infrastructure for repair communities and municipalities, specifically the Urban Resource Centres [26]. Together, they form the backbone of a system shift towards a more sustainable and circular economy. By connecting different activities, the infrastructure serves as a lever for upscaling repair in a systemic and impactful way. The Sharepair project's pilot activity, involving 17 partners, established the foundations of a ORDP. This platform provided a blueprint to support an ecosystem of digital tools and related practices for repair, fostering a collaborative and innovative approach to sustainable consumption and production. Furthermore, the ORDP envisioned to be closely tied to the development of a European infrastructure of Urban Resource Centres, which will provide physical hubs for repair, reuse, and recycling activities. This integrated approach enabled a more comprehensive and effective system for promoting repair and reducing waste, ultimately contributing to a more circular and sustainable economy.

The European data strategy defines a data space as “common data infrastructure and governance frameworks, which facilitates data pooling, access, and sharing” [27]. With in this context, a data space refers to an interoperable environment where data can be discovered, accessed, exchanged, and reused under a shared governance model. The Sharepair’s ORDP followed the same vision to prototype a common data infrastructure that enabled data discovery and interoperability through ORDS and related data standards. Sharepair envisioned the governance of ORDP via a steering board of representatives from ORA, municipalities, and repair communities in a public‑private partnership. This governance model engages multiple stakeholders such as data providers (repair cafés, ORA members, and 3D printing centres), data consumers (citizen repairer, repair cafés, commercial repairers), and government (municipalities) in managing and sustaining the repair data space. The data sharing and management agreement was primarily driven by the project consortium agreement. The ORDP was develop with public‑good model in mind where data are considered free for public repair communities, while value‑added services could be monetised to sustain operations.

## 4 Towards the European Online Repair Platform

To sustain and scale-up to a viable platform, the ORDP needed to be expanded for use in other repair communities and other cities beyond the project consortium. Nonetheless, the Sharepair project shares a common vision with the EORP i.e., to make repair services more accessible and appealing to consumers thereby shifting consumer behaviour from replacement to repair and consequently reducing e-waste. The enabling factors for the success of this shared vision regarding the necessity and utilization of tools and data to support product repairability are as follow.

• **Community Building:** A citizen-centred repair approach cannot function effectively as a digital framework without strong community engagement. This raises the question of how the infrastructure will be developed. Community building focuses on attracting citizens to share their experiences and enhance the data available. Consequently, the development method, which includes pilot projects, is designed to foster communities around repairability within participating organizations, such as Repair Cafés and citizen panel. These initiatives create learning communities that are action oriented.
• **Smart & Circular Cities:** The Sharepair digital infrastructure is aligned with Smart City initiatives that promote a circular economy. Smart cities leverage technology and data to address societal challenges, including the creation of infrastructures that gather relevant data through sensors and citizen science methods. However, there is still progress to be made in circular city policies regarding the use and expansion of data on WEEE, with repair being a vital component. Sharepair advocates for the interoperability of data collection and sharing among participating cities to achieve high levels of data sharing. The network of Smart & Circular Cities is expected to concentrate on policy learning for the preparation of the EORP.
• **Policy Influence:** The “right to repair” is now acknowledged by the European Parliament, but its implementation is still pending. As a result, a coherent “data policy” has become a crucial action point to ensure access to repair information and spare parts for everyone. The EORP will play a significant role in the interaction between repair and consumer organizations and the European Commission services involved in the Circular Economy Action Plan. Standards like ORDS will be a central focus, with the ORA standard needing further refinement and expansion. This standard has the potential to evolve into a European Green Deal data standard, initially by extending its application to independent repair services.

The shared vision of Sharepair and EORP can be undermined by several challenges. These challenges can significantly impact the effectiveness and sustainability of repair efforts across communities.

• **Citizen Engagement and Repair Cafés**: Engaging citizens in repair cafés presents a significant challenge, primarily due to the need for increased awareness about the benefits of repair and the concept of repair cafés themselves. Many individuals may not be familiar with these initiatives, leading to low participation rates. Additionally, the varying levels of expertise among volunteers can hinder effective engagement; structured training programs are often necessary to build confidence and skills among those assisting others. Sustaining interest over time is another hurdle, as attendance can fluctuate, making it essential to implement ongoing activities and incentives that keep the community active and involved.
• **Legal Issues and Insurance:** Legal complexities surrounding liability and insurance pose substantial barriers to the establishment of repair cafés. Volunteers and organizers may hesitate to participate due to concerns about potential accidents or damages that could occur during repair activities. To mitigate these fears, clear guidelines and adequate insurance coverage are crucial. Furthermore, the varying regulations across different countries complicate the creation of a unified vision for repair initiatives, necessitating careful navigation of diverse legal frameworks. Additionally, the sharing of repair knowledge raises questions about intellectual property rights, which must be addressed to foster a collaborative and legally compliant environment.
• **Language Barriers and Cultural Variations:** Language diversity across Europe presents a challenge in effectively communicating the goals and resources of repair initiatives. Developing materials and resources in multiple languages can be resource-intensive and may require partnerships with local organizations to ensure inclusivity. Moreover, cultural attitudes towards repair and sustainability can vary significantly, impacting the acceptance and success of repair cafés in different communities. Understanding these cultural nuances is vital for tailoring approaches that resonate with diverse audiences. Additionally, differences in infrastructure and community needs may affect the effectiveness of repair cafés, necessitating flexibility in adapting models to local contexts while maintaining core principles.

## 5 Recommendations

Based on our experience and recent development across Europe, we discuss the key enablers of the EORP that is foreseen to become operational in 2027 [28].

### 5.1 Digital passports and repair information

One of the key enablers is the *Digital Product Passport* (DPP). It is expected that each DPP will contain information, in the category ``Status, diagnostics, and performance'', enabling circular economy practices like reuse and repair [29]. This information category addresses the demand for usage data and product to determine whether extending the lifetime is possible, based on specialized parameters. Data on the device's health, like its failure diagnostics, physical state, residual lifetime, and usage history, is important because it is valuable data for repair purposes. Data about maintenance records delivers insight into the repair and maintenance, and actions that have happened during a device's life cycle. The DPP can be augmented with repairability information by extending its schema to include spare parts, repair manuals and information, and software or tool requirements. This makes the DPP not just a traceability tool but also a repairability enabler. In practice, consumers can locate repairers or repairability information more efficiently via a new EORP. More specifically, the data set by the manufacturer becomes static part of DPP and any additional data becomes the dynamic part. The dynamic part can include the repair data based on ORDS, as well as sector specific repair details. For instance, for electronics the repair details can include software versions and hardware part serials. In case of industrial machinery, the repair details are can include state of health and cycle counts.

One of the biggest benefits of manufacturing 3D printed parts and components is the ability to more easily trace parts and components throughout their lifetime. Traceability is particularly critical in products for which safety and regulatory compliance are non-negotiable. Integrating the DPP in manufacturing process will enhance traceability of 3D printed parts and components. Therefore, linking the DPP unambiguously with 3D printed parts and components is an essential step, because digital documentation efforts are meaningless if the parts they point to cannot be tracked [30]. For delivering traceability for quality control, it is usually crucial to be capable of tracking down a part in an exact build job and even down to an exact part location in that build. The DPP serves as a complete digital record for individual components, detailing important information like material manufacturing processes, specifications and quality control data, part individual maintenance history, processing parameters, and so on. In summary, a digital passport can significantly contribute to the vision of Sharepair and the EORP by streamlining repair information, enhancing trust, supporting data interoperability, and promoting circular economy practices.

### 5.2 Incentivising product and repair data sharing

Product and repair data sharing is essential for promoting sustainable product reuse and enabling a circular economy; however, it faces significant corporate barriers that discourage companies from sharing relevant data with other companies and citizens. A major concern is the potential loss of competitive advantage, as corporations often view such data as intellectual property and trade secrets. This fear of disclosing proprietary technologies and design processes can lead to concerns over copyright issues [31]. Additionally, companies are reluctant to share repair data due to the risk of losing control over aftermarket services, which are crucial for generating revenue through monopolized after-sales services and spare parts distribution [8]. The business model of many corporations is built around planned obsolescence, where profitability relies on rapid replacement cycles and costly repairs, making them hesitant to disrupt this model by opening access to repair data that could extend product lifetimes [32].

Despite these barriers, there are strong incentives for corporations to share repair data, particularly compliance with regulations such as the ``Right to Repair'' initiatives in the European Union and the United States. By adhering to these regulations, companies can avoid legal risks and position themselves as responsible actors with fair competition practices. For instance, initiatives increasingly require corporations to provide access to repair documentation, spare parts, and diagnostic software, facilitating repair beyond the legal guarantee for products [28]. Repair data sharing can also lead to business model innovation, allowing firms to monetize digital repair ecosystems through subscription-based access to repair data or partnerships with independent repair networks [33]. Furthermore, sharing repair information can enhance corporate reputation by contributing to environmental, social, and governance (ESG) targets, fostering customer loyalty, and differentiating brands in markets increasingly focused on sustainability.

The incentives for sharing repair data, processes, and tools for product manufacturers, intermediatory stakeholders, and repair service providers are multifaceted. Economic benefits and improved service quality are key drivers towards this end [34]. Appropriate incentives and incentive mechanisms can facilitate data sharing by maximizing utility for all parties [35]. Environmental considerations, regulatory preparation, and technological development also motivate manufacturers to engage in repair and remanufacturing initiatives [36]. Citizen-driven repair behaviour is influenced by personal values, beliefs, and attitudes, as well as external factors like access to repair services and economic incentives. To promote repair practices, strategies should focus on creating more repairable products, supporting repair initiatives, and developing skills among repairers [34].

### 5.3 Alignment with common European data spaces

The role of *Common European Data Spaces* is crucial for transforming the repair and reuse practices across Europe and providing the digital backbone for responsible production and consumption [27]. These data spaces can provide the mechanisms for seamless sharing of valuable data which is crucial for scaling up repair businesses and citizen-driven repair efforts. These data spaces range from agriculture to smart cities domains. The Green Deal Data Space (GDDS) [20,21] and European Language Data Space (LDS) [37] are most relevant with the context of Sharepair and EORP.

The SAGE project aims to enable the European Green Deal practices and initiatives by providing a secure federated and interoperable digital infrastructure for integration, governance, and trustful usage of high-value datasets on biodiversity, climate, circular economy, and pollution [20]. This data space should enable the sharing of data regarding the availability of spare parts, repair histories and overall product life cycle. Through its federated and interoperable infrastructure, SAGE enables this data to be shared with transparency and trust across the repair community, from manufacturers to consumers, supporting informed decision-making without requiring centralised data storage [[19], [20], [21],^38^]. This interoperability enhances the overall repairability of products, ease for repairers to access the right parts and guidance regarding all the repair information which contributes to the circular economy by reducing waste and extends product lifespans [4,20,21]. Furthermore, these data spaces function as a bridge between repairers, manufacturers, and consumers, ensuring interoperability and trustworthy data exchange to promote the repair process across Europe [39].

Europe's linguistic diversity, with over 24 official languages and hundreds of regional and minority languages, poses unique challenges and opportunities for repair data sharing and accessibility^4^. The LDS addresses this linguistic diversity by ensuring language related data and services are accessible to everyone [37]. For the EORP, the LDS can play an important role in translating repair instructions, manuals, and guides into multilingual understanding across Europe. LDS services provide repairers and consumers accessibility in common knowledge base, irrespective of location and language. By integrating the SAGE with LDS, community-based repair initiatives as well as professional repair services across different regions can share knowledge and tools with members in more accessible and useful ways. This helps cross-border collaboration for users and community to share repair data, experiences, and solutions without any language barriers creating a more inclusive and collaborative repair ecosystem [2]. This integration with the Green Deal and language data spaces helps to grow the European repair ecosystem in a more systematic and sustainable manner, providing interoperability, accessibility and scalability across the European countries’ digital infrastructure for repair and reuse services. By expanding these data spaces, EORP can become a data-driven repair enabler as envisioned by facilitating sustainability, collaboration, and resource efficiency achieving the goals of the circular economy and sustainable consumption at a pan-European level regardless of language or location [40].

### 5.4 Using artificial intelligence to support repair and reuse

*Artificial Intelligence* (AI) has the potential to revolutionize the product maintenance and repairs in all settings, including commercial repair, community-based repair initiatives and DIY repairers, by tackling important issues in the repair ecosystem, specifically knowledge transfer, diagnostic accuracy, and information accessibility [41]. AI tools and solutions can increase repair productivity by automating diagnostics, optimizing repair procedures, and enhancing inventory control. They can also offer tailored advice depending on the user's abilities and the resources at hand [42]. By anticipating equipment breakdowns, optimizing spare parts inventory, and offering real-time coaching during repairs, AI can increase professional repairers' efficiency while lowering downtime and promoting improved decision-making. AI technologies assist DIY repairers by delivering specific instruction via adaptive intelligent tutoring systems, evaluating repair quality using image analysis tools, and supplying community-driven recommendations derived from successful repair instances. Moreover, AI can enhance the accessibility of repair information by producing documentation from expert demonstrations, streamlining technical manuals, and formulating customized repair instructions tailored to the repairer's expertise and the device's state. Beside the general AI technologies, specific use of computer vision and large language models has potential to significantly improve repairability of products.

*Computer vision* (CV) technologies represent powerful use of AI in repair situations, offering solutions that directly address challenges with visual inspection, damage assessment, and procedural guidance [43]. The ability of use advanced image recognition to identify electrical components, identify damage, and assess spare parts reduces the level of skill needed by DIY repairers while increasing the precision and effectiveness of professional diagnosis [44]. Augmented reality, supported by CV, can project repair instructions onto the device, providing detailed visual instructions particular to the equipment's model and condition [45]. Computer vision can also improve quality assurance in repairs by identifying assembly faults, confirming accurate component placement, and overseeing repair processes in real time to assure adherence to safety standards [46]. Moreover, these technologies also enable the establishment of visual databases for spare parts, allowing repairers to visualize unfamiliar components and immediately acquire identification, sourcing details, compatibility information, and installation assistance. Integrating CV with 3D printing workflows can automatically create replacement parts by assessing damaged components and developing correct digital models, assisting in overcoming spare parts challenges [40].

*Large language models* (LLMs) can help repair knowledge, make it more accessible, and transcend language barriers by analysing technical texts, supporting various languages, and giving intelligent conversational advice [47]. Based on these capabilities, LLMs can automatically write extensive repair manuals based on minimal technical material and translate complex repair procedures into numerous European languages while maintaining technical accuracy. To deliver the best repair techniques for certain device-user combinations, these models are excellent at integrating data from various sources to generate customized repair recommendations. They do this by combining manufacturer requirements, community repair logs, and expert knowledge [48]. LLMs can drive intelligent chatbots that help repair cafés and DIY repairers around-the-clock by providing procedural clarification, safety advice, and troubleshooting support in natural language conversations as part of citizen repair efforts. Additionally, by automatically classifying repair efforts, spotting typical failure patterns, and producing insights that guide both product design enhancements and repair process optimization, LLMs can help extract knowledge from unstructured repair data. Moreover, repair records may be automatically analysed, the probability of a successful repair can be predicted, and repair requests can be matched with the appropriate resources and expertise by integrating LLMs with the ORDS.

## CRediT Author Statement

**Umair ul Hassan**: Conceptualization, Data curation, Funding acquisition, Investigation, Methodology, Project administration, Software, Supervision, Visualization, Writing – original draft, Writing – review and editing; **Prateek Paul**: Investigation, Writing – original draft, Writing – review and editing; **Muhammad Sohaib Ayub**: Conceptualization, Writing – original draft, Writing – review and editing; **Kashif Shaheed**: Conceptualization, Writing – original draft, Writing – review and editing; **Long Hoang**: Investigation, Writing – original draft, Writing – review and editing

## Funding Sources

This work was supported in part by Interreg North West Europe co-funded by the European Union under grant SHAREPAIR and Taighde Éireann - Research Ireland under grants 12/RC/2289_P2 (Insight). For the purpose of Open Access, the author has applied a CC BY public copyright licence to any Author Accepted Manuscript version arising from this submission.

## Ethics Statement

The authors have read and followed the ethical requirements for publication in Data in Brief and confirm that the current work does not involve human subjects, animal experiments, or any data collected from social media platforms.

## Declaration of Generative AI and AI-Assisted Technologies in the Writing Process

As the authors’ native language is not English, they used openly available large language models (GPT-o4 mini, Llama 4 Scout, Gemma 3) to check the language and grammar during the preparation of this work. After using this tool, the authors reviewed and edited the content as needed and take full responsibility for the publication’s content.

## Declaration of Generative AI in Scientific Writing

In this paper, generative AI tools were used solely for language correction, enhancing readability, and improving the clarity of the text. The AI was not involved in data analysis, research design, or the formulation of ideas. All intellectual content and scientific contributions remain the responsibility of the authors.

## Declaration of Competing Interests

The authors declare no competing interests.

## Footnotes

1 https://www.ifixit.com/.

2 https://therestartproject.org/.

3 https://www.reparatur-initiativen.de/.

4 https://european-union.europa.eu/about-eu/eu-in-brief/languages_en.
